# Supplementary material for: It Takes Two to Tango: A Bacterial Biofilm Provides Protection against a Fungus-Feeding Bacterial Predator
Source: Microorganisms. 2021 Jul 23;9(8):1566. doi: 10.3390/microorganisms9081566 (PMC8398733; doi:10.3390/microorganisms9081566)
Supplement: Supplementary file 1 [file microorganisms-09-01566-s001.zip › microorganisms-1303879-supplementary.pdf]

microorganisms

Article

It takes two to tango: a bacterial biofilm provides protection against a fungus feeding bacterial predator

Shubhangi Sharma[1], Stéphane Compant[2], Philipp Franken[1,4], Silke Ruppel[1] and Max-Bernhard Ballhausen[1,3*]

[1]Leibniz Institute of Vegetable and Ornamental Crops, Theodor-Echtermeyer-Weg 1, 14979 Großbeeren, Germany.

[2]AIT Austrian Institute of Technology, Center for Health and Bioresources, Konrad Lorenz Strasse 24, 3430 Tulln, Austria.

[3] Present address: Freie Universität Berlin, Altensteinstraße 6, 14195 Berlin.

[4]Present address: Erfurt Research Centre for Horticultural Crops, University of Applied Sciences Erfurt, Kühnhäuser Straße 101, 99090 Erfurt, Germany and Friedrich Schiller University Jena, Institute of Microbiology, Neugasse 24, 07743 Jena, Germany

*Corresponding author: Max-Bernhard Ballhausen, Freie Universität Berlin, Altensteinstraße 6, 14195 Berlin

Email: m.ballhausen@fu-berlin.de

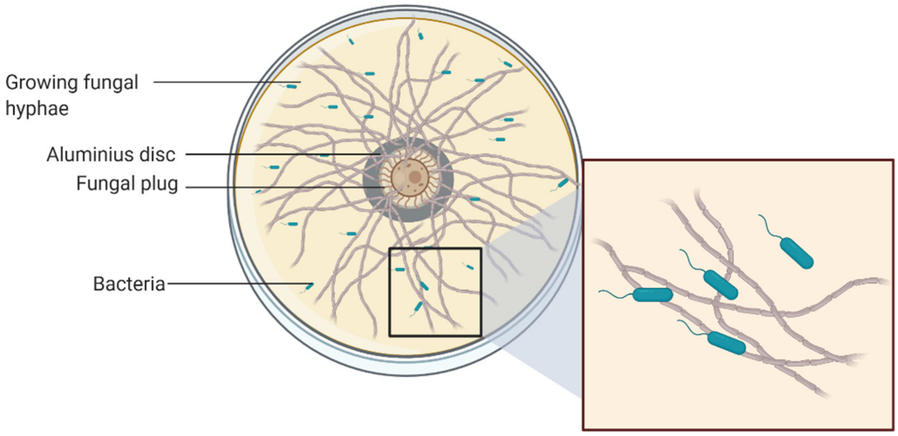

Figure S1: *In vitro* mycophagy assay set up. *Collimonas fungivorans* or *Kosakonia radicincitans* were grown on a Petri dish containing phytagel medium. A fungal plug from an actively growing *Serendipita indica* culture was placed in the center of the Petri dish on an aluminum disc. While expanding radially, growing fungal hyphae were confronted with bacteria.

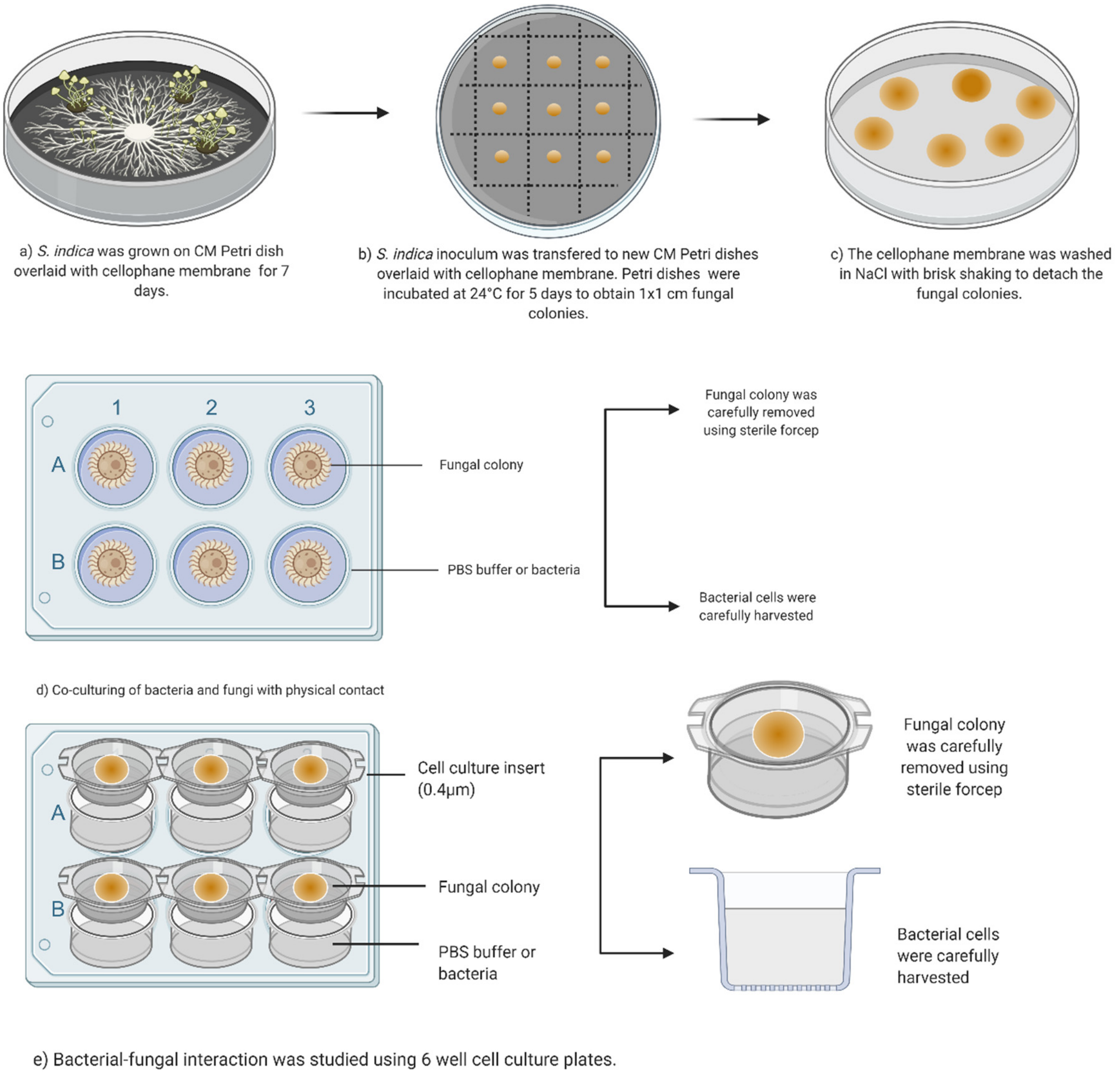

Figure S2: Schematic diagram showing (a-c) culturing of *Serendipita indica* fungal colonies on Petri dishes containing solidified CM overlaid with cellophane membrane and detachment of fungal colonies from the cellophane membrane or (d-e) co-culturing of fungus with *Collimonas fungivorans* or *Kosakonia radicincitans* using 6-well plates with or without cell culture inserts.

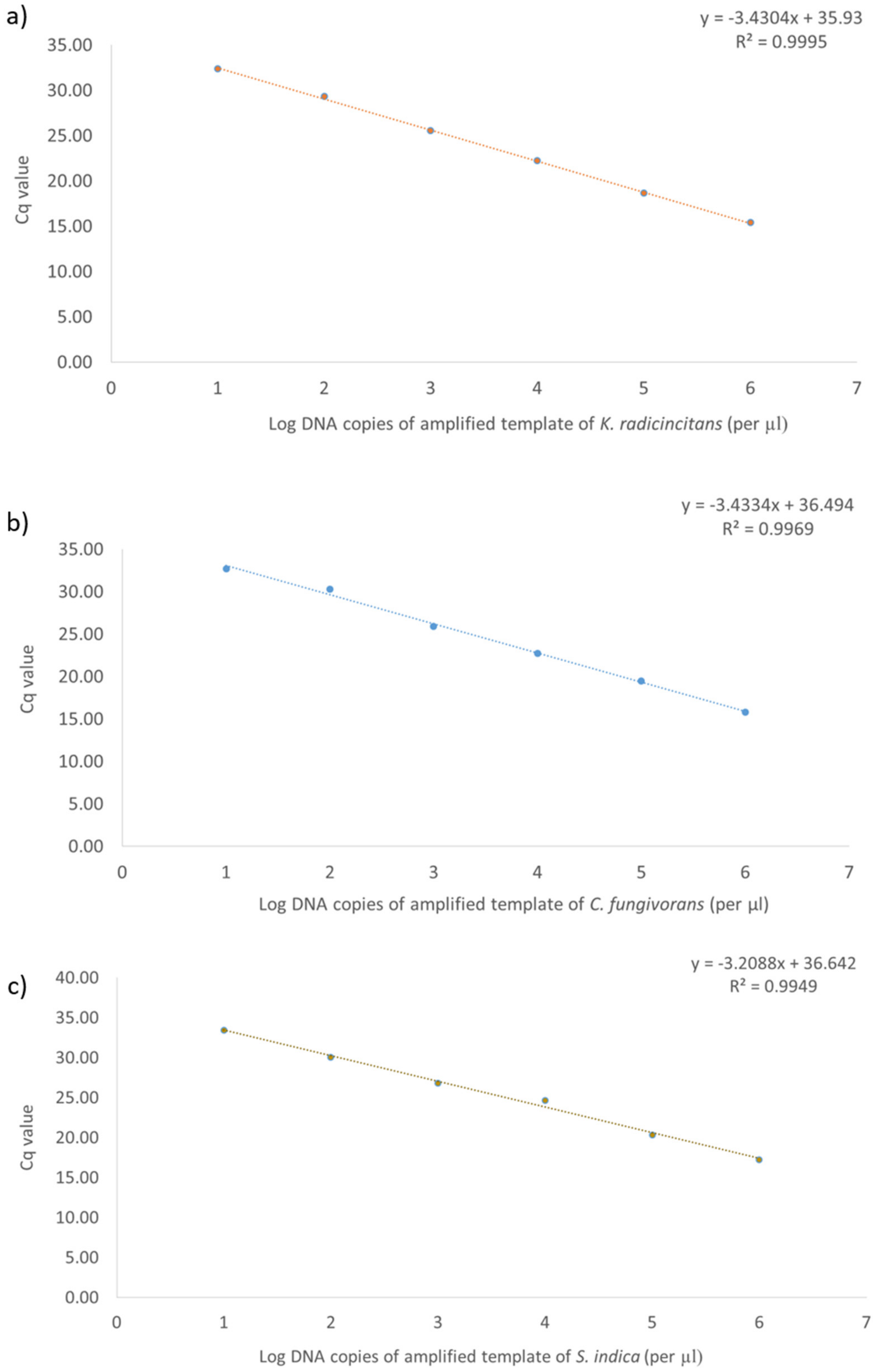

Figure S3: qPCR-generated standard curves of a) *Kosakonia radicincitans* and b) *Collimonas fungivorans* for absolute quantification by TaqMan assay using *Kosakonia* and *Collimonas* specific dual-labelled probes. c) Standard curve of *Serendipita indica*, which was quantified by SYBR green I assay.

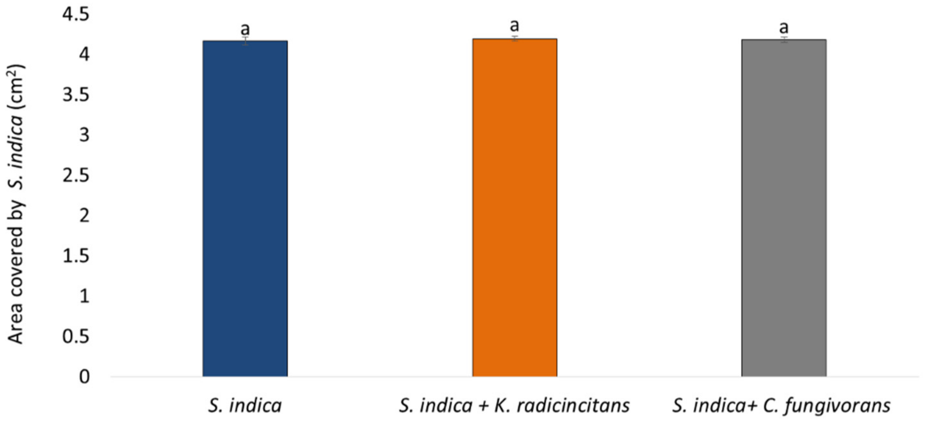

Figure S4: Area of *Serendipita indica* colonies (cm²) after three weeks of growth. *S. indica* was confronted with either *Kosakonia radicincitans* or *Collimonas fungivorans* on water yeast agar plates. Same letters above bars represent no significant differences (P≤0.05) according to one way ANOVA followed by a Tukey's range test. Error bars indicate standard deviation of means of three different replicates.

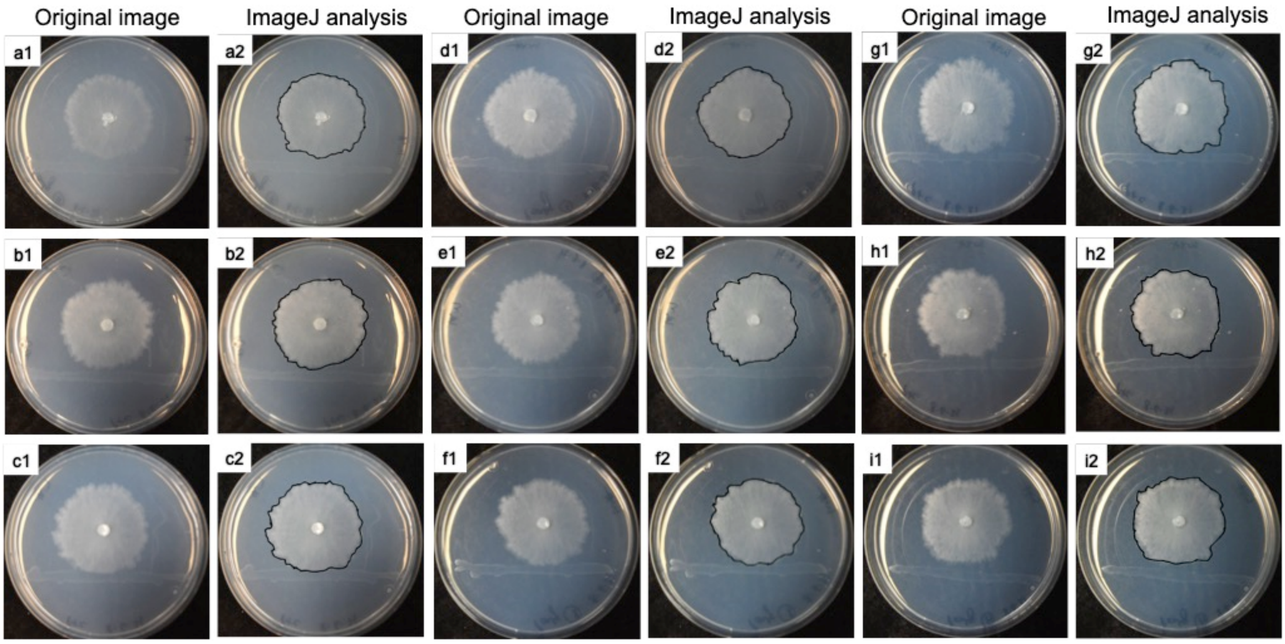

Figure S5: Petri dishes showing the area covered by uninoculated *S. indica* when confronted with the fungus-feeding bacterium *C. fungivorans. (*a1-i1) Original image of the *S. indica* colony growing in the Petri dishes. (a2-i2) Images analyzed by ImageJ software, used to measure the area covered by *S. indica* in the Petri dishes.

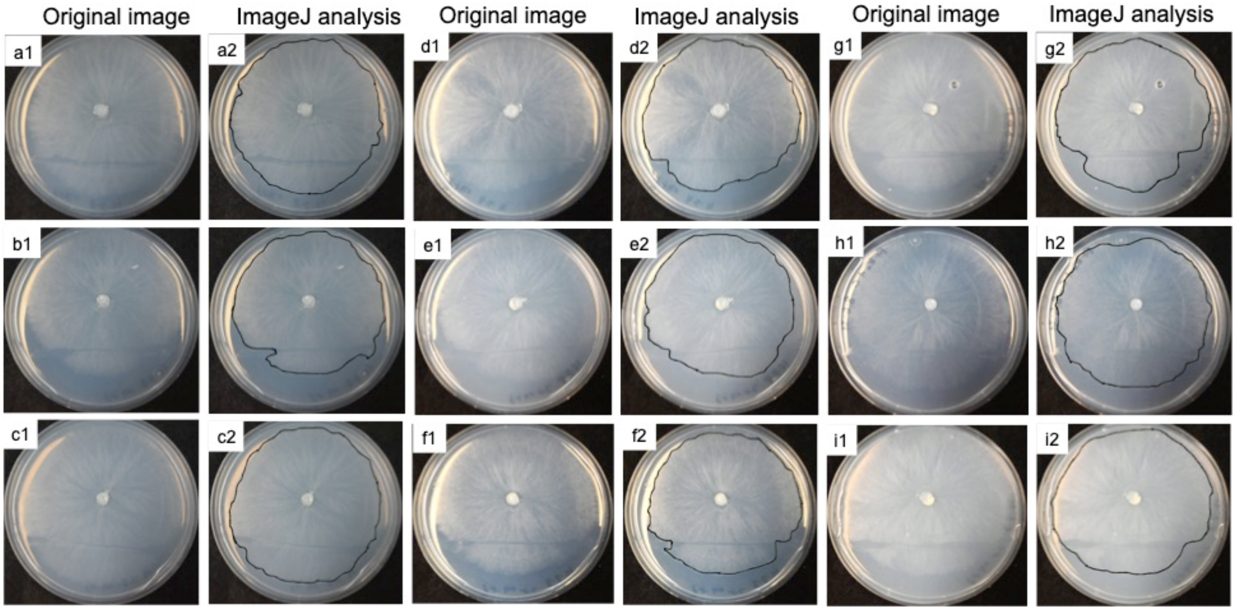

Figure S6: The area covered by *S. indica-K. radicincitans* consortia when confronted with the fungus-feeding bacterium *C. fungivorans.* (a1-i1) Original image of the *S. indica* colony growing on the Petri dishes. (a2-i2) Images analyzed by ImageJ software, used to measure the area covered by *S. indica* on the Petri dishes.

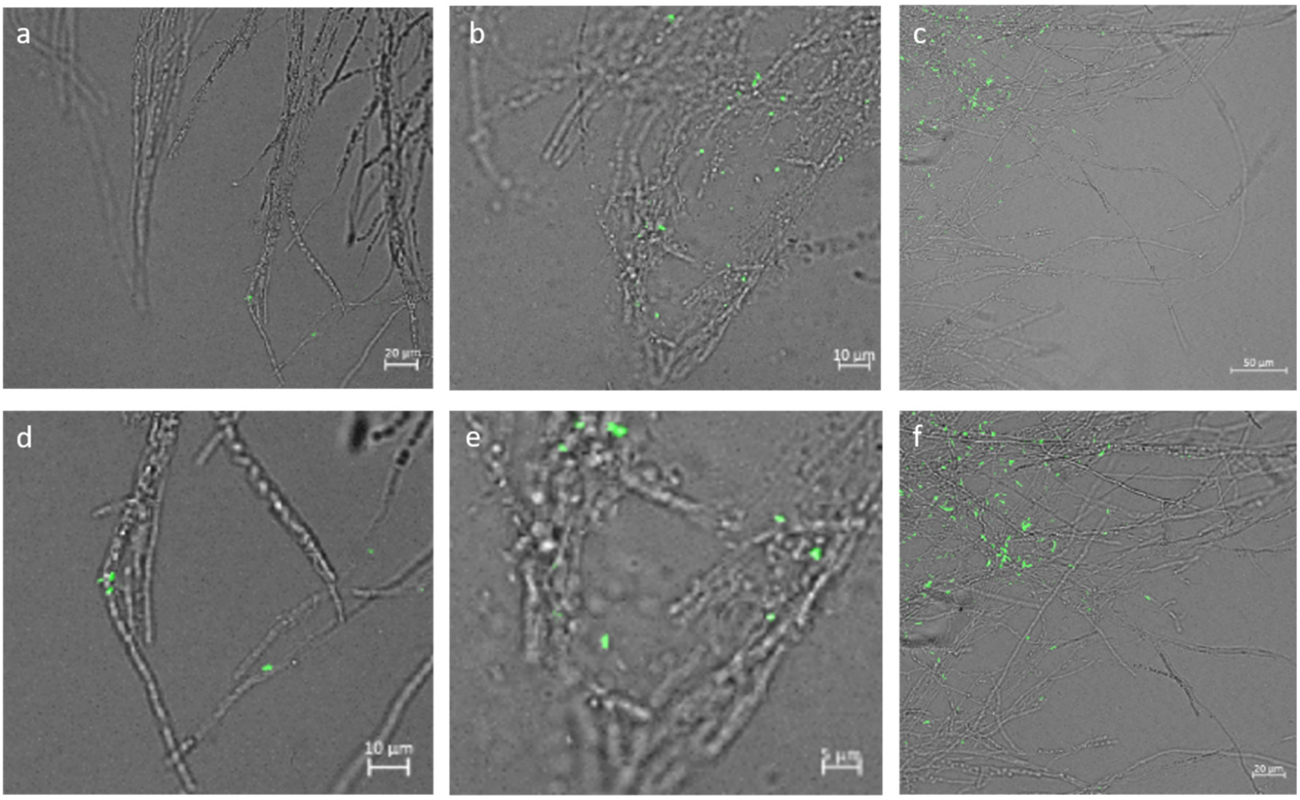

Figure S7: Imaging of microcolonies of *Serendipita indica* co-cultured with *Kosakonia radicincitans* for 72h at 24°C. The colonization of the fungal hyphae by *K. radicincitans* was observed microscopically. (a-c) *K. radicincitans* colonized the hyphae of *S. indica* forming bacterial aggregates 4h post inoculation. (d-e) *K.*

radicincitans aggregates found attached to the hyphal tips of *S. indica*. The GFP strain of *K. radicincitans* was used and *S. indica* is shown in grey scale. Pictures were taken using a confocal microscope at 40x magnification.

Table S1. List of primers and probe sequences used in this study

Primer/Probes	Sequence (5´-3´)	Reference
Tef-f	ACCGTCTTGGGGTTGTATCC	Bütehorn et al., 2000
Tef-r	TCGTCGCTGTCAACAAGATG	Bütehorn et al., 2000
Eddy3for	GTACAGAATCCCGAAGAGATTTGG	Ogawa-Höppener et al., 2007
Eddy3rev	ACTTAACCCAACATCTCACGACA	Ogawa-Höppener et al., 2007
TaqMan probe Sophie	FAM-CGAAAGAAAGCTGTAACACAGG-BHQ	Ogawa-Höppener et al., 2007
519f	CAGCMGCCGCGGTAANWC	Lane et al., 1991
E.radr	CGTGGACTACCAGGGTATCTAATC	Ruppel et al., 2006
E.rad TaqMan probe	[6-FAM] CTCCCCACGCTTTCGCACCTGAGC [BHQ-6-FAM]	Ruppel et al., 2006
